# Investigating the polygenic relationship between heavy cannabis use and schizophrenia in the All of Us Research Program

**DOI:** 10.1017/S0033291725102717

**Published:** 2025-12-17

**Authors:** Isabelle Austin-Zimmerman, Hayley H.A. Thorpe, John J. Meredith, Jibran Y. Khokhar, Tian Ge, Marta Di Forti, Arpana Agrawal, Emma C. Johnson, Sandra Sanchez-Roige

**Affiliations:** 1 Social, Genetic, and Developmental Psychiatry Centre, Institute of Psychiatry, Psychology, and Neuroscience, King’s College London, London, UK; 2 Department of Anatomy and Cell Biology, Schulich School of Medicine and Dentistry, Western University, London, ON, Canada; 3 Department of Psychiatry, University of California San Diego, San Diego, CA, USA; 4 Department of Psychiatry, Harvard Medical School, Boston, MA, USA; 5 Psychiatric and Neurodevelopmental Genetics Unit, Center for Genomic Medicine, Massachusetts General Hospital, Boston, MA, USA; 6 Center for Precision Psychiatry, Department of Psychiatry, Massachusetts General Hospital, Boston, MA, USA; 7 Stanley Center for Psychiatric Research, Broad Institute of MIT and Harvard, Cambridge, MA, USA; 8 South London and Maudsley NHS Foundation Trust, London, UK; 9 Department of Psychiatry, Washington University School of Medicine, St. Louis, MO, USA; 10 Department of Psychiatry, University of California San Diego, La Jolla, CA, USA; 11 Institute for Genomic Medicine, University of California San Diego, La Jolla, CA, USA; 12 Department of Medicine, Division of Genetic Medicine, Vanderbilt University Medical Center, Nashville, TN, USA

**Keywords:** cannabis, GWAS, polygenic risk score, psychosis, schizophrenia

## Abstract

**Background:**

Decades of research have identified a strong association between heavy cannabis use and schizophrenia (SCZ), with evidence of correlated genetic factors. However, many studies on the genetic relationship between cannabis use and psychosis have lacked data on both phenotypes within the same individuals, creating challenges due to unmeasured confounding. We aimed to address this by using multimodal data from the All of Us Research Program, which contains genetic data as well as information on SCZ diagnosis and cannabis use.

**Methods:**

We tested the association between cannabis use disorder (CUD) and SCZ polygenic scores (PGSs) with SCZ and heavy cannabis use. We tested models where both CUD and SCZ PGSs were included as joint predictors of heavy cannabis use and SCZ case status. We defined three sets of cases based on comorbidities: relaxed (assessing for only the primary condition), strict (excluding comorbidity), and dual-comorbidity.

**Results:**

CUD and SCZ polygenic liability were independently associated with heavy cannabis use; the SCZ PGS effect was very modest. In contrast, both SCZ and CUD PGSs were independently associated with SCZ, with independent significant effects of CUD PGS. Polygenic liability to CUD was associated with SCZ in individuals without a documented history of cannabis use, suggesting widespread pleiotropy.

**Conclusions:**

These findings underscore the need for comprehensive models that integrate genetic risk factors for heavy cannabis use to advance our understanding of SCZ etiology.

## Introduction

Chronic cannabis use is associated with greater psychosis risk (Di Forti, Marconi, & Carra, 2015; Di Forti, Quattrone, & Freeman, 2019; Hasan, von Keller, Friemel, et al., 2020; Marconi et al., 2016), psychotic-like experiences (Bagot, Milin, & Kaminer, 2015; Cheng, Parker, Karadag, et al., 2023; Large et al., 2011), and an earlier age of onset for psychotic symptoms (Bagot et al., 2015; Large et al., 2011). Many prior studies have argued that heavy cannabis use increases the risk of psychosis (Di Forti et al., 2019; Hasan et al., 2020; Marconi et al., 2016).

A complementary hypothesis posits that common genetic mechanisms underlie cannabis use and psychosis (Cheng et al., 2023; Johnson, Austin-Zimmerman, Thorpe, et al., 2024; Johnson, Hatoum, Deak, et al., 2021; Khokhar et al., 2018). Genome-wide association studies (GWASs) and twin studies support that schizophrenia (SCZ), the most common psychotic disorder, is heritable (Bigdeli, Genovese, Georgakopoulos, et al., 2020; Trubetskoy, Pardiñas, Qi, et al., 2022; twin-estimated heritability ~ 80%; Owen, Sawa, & Mortensen, 2016) as are lifetime cannabis use (Thorpe, Fontanillas, Meredith, et al., 2025) and cannabis use disorder (CUD; Johnson, Demontis, Thorgeirsson, et al., 2020; Levey, Galimberti, Deak, et al., 2023; twin-estimated heritability ~40–48% and ~51–59%, respectively; Agrawal & Lynskey, 2006; Verweij, Zietsch, & Lynskey, 2010). Cannabis use traits and schizophrenia are positively genetically correlated (*r_g_* range = 0.25–35; Cheng et al., 2023). The most recent Mendelian randomization studies support a bidirectional causal relationship between SCZ and CUD (Elkrief, Lin, Marchi, et al., 2023; Levey et al., 2023), with a greater magnitude of causal effect from CUD to SCZ. Further, with the advent of polygenic scores (PGSs), which aggregate the effect sizes from GWAS across genome-wide variants to quantify genetic liability for a trait, it has been observed that SCZ PGS are associated with psychotic-like experiences in cannabis users (Wainberg, Jacobs, di Forti, & Tripathy, 2021). This indicates that genetic risk for SCZ might make one particularly vulnerable to the psychotogenic effects of cannabis use. Thus, delineating the genetic relationship between cannabis use and SCZ may inform psychosis prevention, particularly in the context of expanding cannabis legalization and accessibility.

Another complicating factor is that while the prevalence of cannabis use and CUD in individuals with SCZ is high (Abush, Ghose, Van Enkevort, et al., 2018; Di Forti et al., 2015, 2019; Hjorthøj, Compton, Starzer, et al., 2023; Hjorthøj, Posselt, & Nordentoft, 2021; Marconi et al., 2016), no GWASs of SCZ account for this co-occurrence. Likewise, GWASs of CUD rarely account for comorbid psychosis unless such conditions are exclusionary criteria for study recruitment. Thus, it is plausible that the genetic correlation between SCZ and cannabis use/CUD is partially confounded by their co-occurrence in the population.

In the present study, we availed of a unique opportunity to disarticulate the SCZ–cannabis comorbidity by leveraging the multimodal data of the National Institute of Health’s *All of Us* (AoU) Research Program, which provides phenotypic data relevant to cannabis and SCZ, as well as genetic data, for up to ~250,000 participants in the version 7 release. Using data from prior well-powered GWASs, we examined the association between CUD (Levey et al., 2023) and SCZ (Bigdeli et al., 2020; Trubetskoy et al., 2022) PGS, and cannabis-related traits and SCZ in the AoU cohort. We hypothesized that polygenic liability to both CUD and SCZ would exert independent effects on cannabis use and SCZ. However, we also expected that the associations would be attenuated in the absence of the comorbid condition (e.g., SCZ PGS would not strongly relate to cannabis-related traits without comorbid psychosis).

## Methods

### Participants

Participants enrolled in AoU (v7) were included in our analysis. AoU is a diverse database of electronic health records (EHR), survey responses, physical measurements, and whole-genome sequencing (WGS) data from blood or saliva samples; the v7 release included data for over ~250,000 adults in the United States. For details on recruitment and genomic data, please see previous publications from the AoU Research Program (All of Us Research Program Investigators et al., 2019; All of Us Research Program Investigators, 2024; Ramirez, Sulieman, Schlueter, et al., 2022).

Unrelated participants who were assigned male or female at birth, had applicable EHR and/or survey-level data, had WGS data, and whose genomes were statistically correlated with the genomes of European (EUR) or African (AFR) reference populations were included in the study. Genetic similarity was based on principal components (PCs) provided by AoU. Related participants (kinship score > 0.1) were removed using a relatedness flagged sample list provided by AoU kinship estimation that minimized the number of samples needing to be removed (All of Us Research Program Investigators, 2024).

Heavy cannabis use and SCZ were derived using multimodal data, including diagnostic codes (SNOMED Clinical Terms), survey data, and prescription medication records (Table 1). We defined heavy cannabis use as anyone with a diagnosis of CUD, as well as anyone who reported daily cannabis use within the last 3 months. We included daily cannabis use based on its strong correlations with CUD (Thorpe et al., 2025) and with SCZ (Di Forti et al., 2015; Marconi et al., 2016). We defined SCZ as anyone with SCZ based on diagnostic codes and/or survey data. Participants were excluded from the control groups if they had a record of antipsychotic prescriptions. See Supplementary Figure 1 for a participant inclusion flowchart.

**Table 1.** All of Us concept codes used to assign binary identifiers for schizophrenia, heavy cannabis use, heavy tobacco smoking, and antipsychotic medication statuses. Participants across different Concept IDs may overlap. See Supplementary Figure 1 for sample details.

Phenotype	Concept ID	Source	*N*_cases_	Concept
Schizophrenia	435783	Conditions	2,445	Schizophrenia
	43530520	Personal and Family Health Survey	440	About how old were you when you were first told you had schizophrenia?
	43528847	Personal and Family Health Survey	335	Are you currently prescribed medications and/or receiving treatment for schizophrenia?
	43530362	Personal and Family Health Survey	347	Are you still seeing a doctor or health care provider for schizophrenia?
Heavy cannabis use	1585650	Lifestyle Survey	3,954	In the PAST 3 MONTHS, how often have you used Marijuana (cannabis, pot, grass, hash, weed, etc.)? [Response ‘Daily use’]
	434327	Conditions	3,712	Cannabis abuse
	440387	Conditions	934	Cannabis dependence
	440996	Conditions	213	Cannabis dependence in remission
	433452	Conditions	143	Cannabis dependence, continuous
	437838	Conditions	26	Cannabis dependence, episodic
	4323639	Conditions	53	Cannabis misuse
	4103419	Conditions	0	Nondependent cannabis abuse
	435231	Conditions	122	Nondependent cannabis abuse in remission
	434019	Conditions	<20	Nondependent cannabis abuse, continuous
	434328	Conditions	<20	Nondependent cannabis abuse, episodic
Heavy tobacco smoking	1586162	Lifestyle Survey	7,373	On average, over the entire time that you smoked, how many cigarettes did you smoke each day? (There are 20 cigarettes in a pack.) [Response >20]
Antipsychotic prescription	21604490	Drugs	31,511	Antipsychotics

We classified participants into different case/control groups (Supplementary Figure 1). First, we used ‘relaxed’ definitions that assigned individuals to case status regardless of comorbidity (e.g., SCZ with or without a history of heavy cannabis use). Second, to account for the comorbid phenotypic manifestation of SCZ and heavy cannabis use, we created ‘strict’ definitions that excluded comorbidities (i.e., cases with heavy cannabis use histories that excluded those with a SCZ diagnosis; SCZ cases excluding those with a history of heavy cannabis use). Finally, we also defined a comorbid case group, which included those with *both* a history of heavy cannabis use *and* a SCZ diagnosis.

### Polygenic analysis

All analyses were conducted using the AoU Researcher Workbench cloud computing environment. Single-nucleotide polymorphisms (SNPs) were curated using the AoU short-read WGS Allele Count/Allele Frequency call set for each ancestral subpopulation, and further filtered to only biallelic SNPs present in HapMap3 from the EUR and AFR 1,000 Genomes Linkage Disequilibrium Scores. Cohort-specific PGSs were calculated using polygenic risk score continuous shrinkage (PRS-CS) ‘auto’ v1.1.0 (Ge et al., 2019) based on SNPs identified by publicly available GWAS summary statistics of SCZ (AFR *N*_cases_ = 7,509, *N*_controls_ = 8,337; Bigdeli et al., 2020; EUR *N*_cases_ = 67,390, *N*_controls_ = 94,015; Trubetskoy et al., 2022) and CUD (AFR *N*_cases_ = 19,065, *N*_controls_ = 104,143; EUR *N*_cases_ = 42,281, *N*_controls_ = 843,744; Levey et al., 2023) and that intersected with SNPs in the AoU database. The ‘auto’ mode of PRS-CS allows the shrinkage parameter (phi) to be learned from the data automatically. We used the default values for Markov Chain Monte Carlo (1,000) and burn-in (500) iterations, which are recommended for stable posterior estimation. SCZ and CUD PGSs were created from up to 973,634 SNPs using the allelic-scoring function, *score*, in PLINK (v1.9; Ge et al., 2019).

#### Statistical analyses

We examined the independent and joint effect of CUD and SCZ PGSs on both heavy cannabis use and SCZ case status from unrelated individuals (All of Us Research Program Investigators, 2024) using various case/control groups based on relaxed, strict, and comorbid definitions (Supplementary Figure 1). All models were adjusted for age, sex, and the first 10 ancestry-specific genomic PCs. Tobacco smoking is also prevalent among those with cannabis use and SCZ (Agrawal, Budney, & Lynskey, 2012; Ziedonis, Hitsman, Beckham, et al., 2008); we performed sensitivity analyses using heavy tobacco smoking as a covariate.

Liability scale *R* ^2^ was calculated according to Lee et al (Lee, Goddard, Wray, & Visscher, 2012) using the R package *fmsb* (v0.7.6), the *NagelkerkeR2* function, and the estimated population-level prevalence of 0.90% for SCZ (Perälä, Suvisaari, Saarni, et al., 2007) and 6.27% for CUD (Hasin, Kerridge, Saha, et al., 2016) in US adults.

## Results

Participant demographics are described in Supplementary Table 1. For the case/control composition, see Supplementary Figure 1, and for the distribution of PGSs across groups, see Supplementary Figures 2 and 3.

*CUD-PGS and SCZ-PGS associations with heavy cannabis use:*

**Figure 1.**
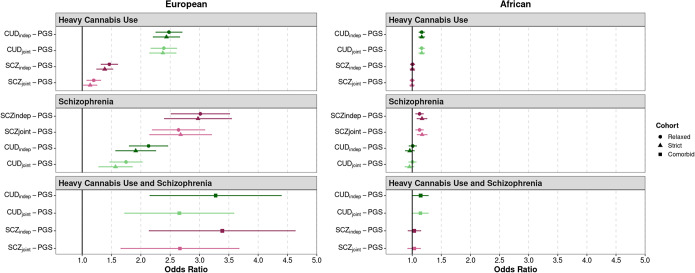
CUD and schizophrenia (SCZ) polygenic score associations with heavy cannabis use and schizophrenia. Associations shown for European and African samples under Relaxed (cases only screened for the primary outcome), Strict (cases with comorbid schizophrenia or heavy cannabis use removed), and comorbid (including both schizophrenia and heavy cannabis use) case definitions. Analyses conducted with CUD and SCZ PGSs considered independently (indep) or jointly (joint).

*Relaxed case definitions.* In the EUR sample, both CUD-PGS and SCZ-PGS were significantly associated with heavy cannabis use when modeled independently, with a larger magnitude of effect for CUD-PGS, as expected (CUD_ind_-PGS odds ratio [OR] = 2.479, 95% confidence interval [CI]: 2.267–2.711; SCZ_ind_-PGS OR = 1.464, 95% CI: 1.33–1.611; Table 2 and Figure 1). In the model including both PGSs, CUD-PGS remained strongly associated with heavy cannabis use (CUD_joint_-PGS OR = 2.391, 95% CI: 2.182–2.620), with only a 3.51% reduction in the effect size when modeled alongside SCZ-PGS, while SCZ-PGS retained a significant but attenuated effect (SCZ_joint_-PGS OR = 1.198, 95% CI: 1.086–1.321), with an 18.24% reduction in the effect size compared to modeling SCZ-PGS alone.

**Table 2.** Summary of polygenic associations with heavy cannabis use and schizophrenia across various case group definitions. a: analyses include all heavy cannabis users regardless of schizophrenia status or all schizophrenia cases regardless of heavy cannabis use patterns (“relaxed”) or either exclude all comorbid cases or include only comorbid cases (“strict”). b: ind=Independently modelled; joint=both PGSs modelled jointly.

Case group definition	Population	PGS	Outcome	OR	95% CI	*P*
*Heavy cannabis use*						
*Relaxed^a^*	EUR	CUD_ind_^b^	Heavy cannabis use (*N*_case_ = 3,122; *N*_control_ = 91,500)	2.479	2.267:2.711	3.07 × 10^–88^
		SCZ_ind_		1.464	1.33:1.611	5.74 × 10^–15^
		CUD_joint_^b^		2.391	2.182:2.620	6.87 × 10^–78^
		SCZ_joint_		1.198	1.086:1.321	3.15 × 10^–04^
	AFR	CUD_ind_	Heavy cannabis use (*N*_case_ = 4,492; *N*_control_ = 36,940)	1.164	1.118:1.210	5.73 × 10^–14^
		SCZ_ind_		1.006	0.971:1.043	7.34 × 10^–01^
		CUD_joint_		1.164	1.118:1.210	6.07 × 10^–14^
		SCZ_joint_		1.000	0.964:1.036	9.80 × 10^–01^
*Strict^a^*	EUR	CUD_ind_	Heavy cannabis use (*N*_case_ = 2,920; *N*_control_ = 91,248)	2.436	2.222:2.672	5.70 × 10^–80^
		SCZ_ind_		1.385	1.255:1.529	1.01 × 10^–10^
		CUD_joint_		2.375	2.162:2.610	2.95 × 10^–72^
		SCZ_joint_		1.136	1.027:1.257	1.30 × 10^–02^
	AFR	CUD_ind_	Heavy cannabis use (*N*_case_ = 4,014; *N*_control_ = 36,634)	1.163	1.116:1.213	8.98 × 10^–13^
		SCZ_ind_		1.004	0.967:1.042	8.47 × 10^–01^
		CUD_joint_		1.164	1.116:1.213	8.99 × 10^–13^
		SCZ_joint_		0.997	0.960:1.035	8.67 × 10^–01^
*Schizophrenia*						
*Relaxed*	EUR	SCZ_ind_	Schizophrenia (*N*_case_ = 1,013; *N*_control_ = 98,008)	3.014	2.555:3.555	3.84 × 10^–39^
		CUD_ind_		2.131	1.828:2.485	4.22 × 10^–22^
		SCZ_joint_		2.643	2.232:3.131	2.21 × 10^–29^
		CUD_joint_		1.748	1.494:2.045	3.32 × 10^–12^
	AFR	SCZ_ind_	Schizophrenia (*N*_case_ = 1,627; *N*_control_ = 43,631)	1.127	1.064:1.193	4.53 × 10^–5^
		CUD_ind_		1.009	0.948:1.074	7.72 × 10^–01^
		SCZ_joint_		1.127	1.064:1.193	4.69 × 10^–05^
		CUD_joint_		1.005	0.944:1.069	8.87 × 10^–01^
*Strict*	EUR	SCZ_ind_	Schizophrenia (*N*_case_ = 753; *N*_control_ = 91,248)	2.975	2.455:3.603	7.93 × 10^–29^
		CUD_ind_		1.913	1.601:2.285	8.88 × 10^–13^
		SCZ_joint_		2.679	2.201:3.260	8.08 × 10^–23^
		CUD_joint_		1.568	1.307:1.881	1.27 × 10^–6^
	AFR	SCZ_ind_	Schizophrenia (*N*_case_ = 1,010; *N*_control_ = 36,634)	1.168	1.087:1.256	2.52 × 10^–05^
		CUD_ind_		0.959	0.886:1.038	3.00 × 10^–01^
		SCZ_joint_		1.170	1.088:1.258	2.09 × 10^–05^
		CUD_joint_		0.953	0.881:1.031	2.31 × 10^–01^
*Comorbid*	EUR	CUD_ind_	Comorbid heavy cannabis use and schizophrenia (*N*_case_ = 202, *N*_control_ = 91,248)	3.275	2.328:4.608	9.69 × 10^–12^
		SCZ_ind_		3.387	2.346:4.889	7.38 × 10^–11^
		CUD_joint_		2.655	1.871:3.768	4.56 × 10^–08^
		SCZ_joint_		2.667	1.829:3.888	3.39 × 10^–07^
	AFR	CUD_ind_	Comorbid heavy cannabis use and schizophrenia (*N*_case_ = 478; *N*_control_ = 36,634)	1.144	1.021:1.282	2.03 × 10^–02^
		SCZ_ind_		1.039	0.936:1.152	4.74 × 10^–01^
		CUD_joint_		1.143	1.019:1.281	2.20 × 10^–02^
		SCZ_joint_		1.033	0.931:1.146	5.41 × 10^–01^

In the AFR sample, CUD-PGS was also associated with heavy cannabis use, both when modeled independently (CUD_ind_-PGS OR = 1.164, 95% CI: 1.118–1.210) and when modeled jointly with SCZ-PGS (CUD_joint_-PGS OR = 1.164, 95% CI: 1.118–1.210; Table 2 and Figure 1). SCZ-PGS was not significantly associated with heavy cannabis use when modeled independently (SCZ_ind_-PGS OR = 1.006, 95% CI: 0.971–1.043) nor when modeled jointly (SCZ_joint_-PGS OR = 1.000, 95% CI: 0.964–1.036).

*Strict case definitions.* We next investigated associations between CUD-PGS and SCZ-PGS and heavy cannabis use when all cases with comorbid SCZ were excluded. In the EUR sample, CUD-PGS remained significantly associated with heavy cannabis use (CUD_ind_-PGS OR = 2.436, 95% CI: 2.222–2.672) while SCZ-PGS showed a significant but somewhat attenuated association (SCZ_ind_-PGS OR = 1.385, 95% CI: 1.255–1.529). When both PGSs were included, CUD-PGS was associated with heavy cannabis use (CUD_joint_-PGS OR = 2.375, 95% CI: 2.162–2.610), while the SCZ-PGS association was further attenuated (OR = 1.136, 95% CI: 1.027–1.257, *p* = 0.013).

In the AFR sample, CUD-PGS was also associated with heavy cannabis use (CUD_ind_-PGS OR = 1.163, 95% CI: 1.116–1.213; Table 2 and Figure 1). SCZ-PGS was not significantly associated with heavy cannabis use when modeled independently (SCZ_ind_-PGS OR = 1.004, 95% CI: 0.967–1.042) or when modeled jointly (SCZ_joint_-PGS OR = 0.997, 95% CI: 0.960–1.035).

*CUD-PGS and SCZ-PGS associations with SCZ:*

*Relaxed case definitions.* In the EUR sample, the SCZ-PGS and CUD-PGS were significantly associated with SCZ when modeled independently, with a larger magnitude of effect for the within-trait association (SCZ_ind_-PGS OR = 3.014, 95% CI: 2.555–3.555; CUD_ind_-PGS OR = 2.131, 95% CI: 1.828–2.485). The inclusion of both PGSs in a joint model resulted in a slight reduction in effect size for both PGSs (SCZ_joint_-PGS OR = 2.643, 95% CI: 2.232–3.131; CUD_joint_-PGS OR = 1.748, 95% CI: 1.494–2.045; Table 2 and Figure 1).

In the AFR sample, the SCZ-PGS was associated with SCZ, both when modeled independently (SCZ_ind_-PGS OR = 1.127, 95% CI: 1.064–1.193) and when modeled jointly (SCZ_joint_-PGS OR = 1.127, 95% CI: 1.064–1.193). CUD-PGS were not associated with SCZ when modeled independently (CUD_ind_-PGS OR = 1.009, 95% CI: 0.948–1.074) nor when modeled jointly (CUD_joint_-PGS OR = 1.005, 95% CI: 0.944–1.069; Table 2 and Figure 1).

*Strict case definitions.* We considered whether the explanatory power of SCZ-PGS and CUD-PGS would differ when all SCZ cases with a history of heavy cannabis use were excluded (Table 2 and Figure 1). Among EUR participants, SCZ-PGS was highly associated with SCZ in both the independent and joint models (SCZ_ind_-PGS OR = 2.975, 95% CI: 2.455–3.603; SCZ_joint_-PGS OR = 2.679, 95% CI: 2.201–3.260), while CUD-PGS had a smaller but significant effect (CUD_ind_-PGS OR = 1.913, 95% CI: 1.601–2.285; CUD_joint_-PGS OR = 1.568, 95% CI: 1.307–1.881). In the AFR sample, SCZ-PGS was significantly associated with SCZ (SCZ_ind_-PGS OR = 1.168, 95% CI: 1.087–1.256; SCZ_joint_-PGS OR = 1.170, 95% CI: 1.088–1.258). CUD-PGS had a weaker and nonsignificant effect (CUD_ind_-PGS OR = 0.959, 95% CI: 0.886–1.038; CUD_joint_-PGS OR = 0.953, 95% CI: 0.881–1.031).

*CUD-PGS and SCZ-PGS associations with comorbid heavy cannabis use and* SCZ*:*

Finally, we considered the explanatory value of both SCZ-PGS and CUD-PGS on cases with comorbid SCZ and heavy cannabis use (Table 2 and Figure 1). In the EUR sample, both PGSs were associated with these comorbid cases when modeled independently (CUD_ind_-PGS OR = 3.275, 95% CI: 2.328–4.608; SCZ_ind_-PGS OR = 3.387, 95% CI: 2.346–4.889), and the effect sizes were slightly reduced for both PGSs when modeled jointly (SCZ_joint_-PGS OR = 2.667, 95% CI: 1.829–3.888; CUD_joint_-PGS OR = 2.655, 95% CI: 1.871–3.768). In the AFR sample, we observed a similar pattern, although the SCZ-PGS was not significantly associated with these comorbid cases in either the independent or joint models (SCZ_ind_-PGS OR = 1.039, 95% CI: 0.936–1.152; SCZ_joint_-PGS OR = 1.033, 95% CI: 0.931–1.146; CUD_ind_-PGS OR = 1.144, 95% CI: 1.021–1.282; CUD_joint_-PGS OR = 1.143, 95% CI: 1.019–1.281).

## Discussion

**Figure 2.**
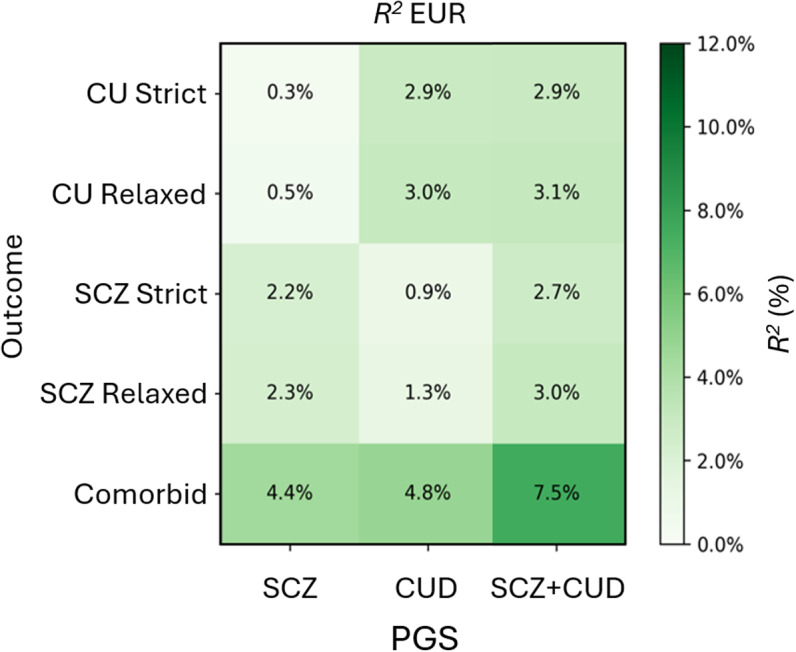
Liability-scale *R^2^* for PGS associations with heavy cannabis use (CU) and schizophrenia (SCZ) in EUR estimated from a population-level prevalence of 0.90% for schizophrenia (Perälä, Suvisaari, Saarni, et al., 2007) and 6.27% for CUD (Hasin, Kerridge, Saha, et al., 2016).

Decades of research have identified a strong association between heavy cannabis use and SCZ, with evidence of correlated genetic factors. However, the genetic characterization of this comorbidity remains incomplete because prior studies have not fully accounted for the genetic correlation between CUD and SCZ or the phenotypic co-occurrence of these traits in target samples. In this study, we modeled CUD-PGS and SCZ-PGS jointly and found that while SCZ-PGS explained little additional variance in heavy cannabis use beyond CUD-PGS (0.1% increase), CUD-PGS accounted for greater (although still modest) variance explained for SCZ (0.7% increase beyond the SCZ-PGS). We hypothesized that more stringent case definitions would improve specificity and help delineate the extent to which underlying genetic risk contributed to CUD/SCZ case status. However, we showed that there was little difference in the effect sizes between the relaxed (i.e., case status regardless of comorbidity) versus strict (i.e., case status excluding comorbidity) and comorbid definitions (Figure 1). For instance, the association between CUD-PGS and both heavy cannabis use and SCZ, regardless of their comorbidity, was statistically equivalent (range: 1.91–3.28). Even though CIs were wide, outcome associations between CUD and SCZ PGSs were notably higher for the comorbid definition (*R* ^2^ up to 7.5%) compared to the relaxed and strict definitions (~3%; Figure 2). These findings have important implications for SCZ etiology, and demonstrate that CUD genetic liability is associated with SCZ risk even in individuals who do not report heavy cannabis use. Taken together, these findings suggest that prior genetic correlations between CUD and SCZ are partially due to true horizontal pleiotropy rather than confounding due to co-occurring diagnoses. In other words, regardless of comorbidity, the etiologies of CUD and SCZ are partially due to the same genetic pathways.

When investigating the relationships between CUD-PGS and SCZ-PGS with heavy cannabis use status using relaxed case definitions, we identified that, as expected, within-trait PGS associations (i.e., CUD-PGS and heavy cannabis use) provided the strongest explanatory power (3% in the EUR sample and 0.3% in the AFR sample). Excluding individuals with SCZ case status did not increase explanatory power (2.9% in the EUR sample and 0.3% in the AFR sample). We hypothesize that this may be the case because many contributing cohorts in the CUD GWAS have already been excluded for psychotic disorders. In contrast, SCZ-PGS showed a weak association with heavy cannabis use in the relaxed case analysis, and this was further reduced when CUD-PGS was included in joint models. This finding suggests that previous reports of SCZ-PGS predicting cannabis use may partially result from unmeasured comorbidity between SCZ risk and cannabis use (Elkrief et al., 2023; Hiemstra, Nelemans, Branje, et al., 2018; Verweij, Abdellaoui, Nivard, et al., 2017).

When investigating the relationship between CUD-PGS and SCZ-PGS and SCZ status, SCZ-PGS was associated with SCZ regardless of case definition (2.2–2.33% in the EUR sample and 0.5–0.6% in the AFR sample). Similarly, CUD-PGS was associated with SCZ regardless of case definition (0.9–1.3% in the EUR sample and 0.05% in the AFR sample). When modeled jointly, CUD-PGS explained substantial phenotypic variance of SCZ above and beyond that of SCZ-PGS (28.14% increase). While it is possible that individuals with higher CUD-PGS are more likely to use cannabis, and that cannabis use could increase SCZ risk, our findings suggest that the association between CUD-PGS and SCZ is not solely explained by cannabis use. Instead, this reflects potential genetic liability shared between the two conditions, consistent with horizontal pleiotropy (Johnson et al., 2021). Mendelian randomization studies suggest bidirectional causal effects between CUD and SCZ (Johnson et al., 2020; Levey et al., 2023; Lin, Pries, Sarac, et al., 2022). Although this study was not designed to interrogate the causal nature of the observed associations, future analyses could build upon our work to calculate PGSs that are derived from multivariate methods (i.e., genomic structural equation modeling) that parse CUD and SCZ genetics, or select variants based on Mendelian randomization results (Garfield & Anderson, 2024). These findings also caution future Mendelian randomization studies to carefully estimate and account for horizontal pleiotropy (e.g., MR-PRESSO; Verbanck, Chen, Neale, & Do, 2018) when investigating the causal relationships between cannabis use and SCZ.

There are limitations worth noting. PGSs are inherently limited by the GWAS data used to build them. While we used PRS-CSx (Ge et al., 2019) to improve power by leveraging GWAS findings from both EUR and AFR populations (Supplementary Table 3), differences in predictive power of PGS in the AFR sample are likely driven by poor PGS generalizability rather than true biological or environmental effects (Atkinson, Bianchi, Ye, et al., 2022). Even in the EUR sample, it is important to note that genetic liability for these traits explains a relatively small proportion of the variance in both outcomes. Therefore, these PGSs are not yet clinically meaningful. Moreover, CUD is genetically correlated with a variety of other behaviors (e.g., depressed mood, executive functioning, and impulsivity; Johnson et al., 2020; Levey et al., 2023), which may co-occur with SCZ, and thus CUD-PGS may have indexed pleiotropic risk that extends beyond associations with heavy cannabis use. Of note, while tobacco use is both correlated with CUD and SCZ, and may be partly responsible for this residual association, adjusting for tobacco use did not alter our findings (Supplementary Table 3; Johnson et al., 2024). Our reliance on EHR and/or self-report data may underestimate the true prevalence of cannabis and tobacco use disorders in our cohort (e.g., ~6.7% or past-year CUD and ~8.5% for nicotine dependence in the general US population vs. ~2.0 and ~6.2% in our sample, respectively). Similar underreporting has been noted in prior studies using AoU data (Barr, Bigdeli, & Meyers, 2022). In the UK Biobank, participants with SCZ diagnoses have been observed to have less severe symptomatology and better health outcomes than clinically ascertained samples, attributable to volunteer bias (Legge, Pardiñas, Woolway, et al., 2024). It may be that participants with cannabis or tobacco use disorders in AoU are similarly less severe than clinical cohorts. We also noted higher rates of SCZ in the AFR ancestry sample than in the EUR ancestry sample. Similar patterns have been reported previously (Schwartz & Blankenship, 2014) and may reflect clinician bias or differential access to healthcare. However, because AoU is not a representative sample of the US population, further epidemiological studies are needed to accurately characterize SCZ prevalence across ancestries. Finally, the SCZ-PGS association with heavy cannabis use may be due to undetected CUD in SCZ GWAS. The same is true for CUD GWAS, but less apparent due to the low SCZ prevalence. Without accounting for phenotypic overlap in the original GWASs, it will be difficult to confirm the extent to which this explains cross-trait associations.

In conclusion, we find compelling evidence of common underlying genetic mechanisms between CUD and SCZ. Future studies with more granular phenotyping will be essential to better understand the true extent of this putative horizontal pleiotropy.

## Supporting information

Austin-Zimmerman et al. supplementary material 1 Austin-Zimmerman et al. supplementary material

Austin-Zimmerman et al. supplementary material 2 Austin-Zimmerman et al. supplementary material

## Data Availability

The AoU workspace used for this project will be made available upon request to registered and eligible AoU researchers through the AoU Research Workbench.

## Supplementary material

The supplementary material for this article can be found at http://doi.org/10.1017/S0033291725102717.
